# Capsaicin suppresses metastasis in tongue squamous cell carcinoma by reversing epithelial-mesenchymal transition via the AMPK/HIF-1α/Claudin-1 axis

**DOI:** 10.1186/s13062-026-00938-6

**Published:** 2026-08-11

**Authors:** Zhuang Li, Qiwei Zhao, Yu Qi, Yu Wang, Long Ding, Shuqin Long, Qiushi Cao, Yaqin Sun, Rui Sun, Xiaohong Guo

**Affiliations:** 1 Department of Basic Medicine, Hubei University of Chinese Medicine, No. 16, West Huangjiahu Road, Wuhan, Hubei 430065 P.R. China; 2 Center of Traditional Chinese Medicine Modernization for Liver Diseases, Hubei University of Chinese Medicine, Wuhan, Hubei 430065 P.R. China; 3 Department of Stomatology, Shanxi Bethune Hospital, Shanxi Academy of Medical Sciences, Tongji Shanxi Hospital, Third Hospital of Shanxi Medical University, No. 99, Longcheng Street, Taiyuan, Shanxi 030032 P.R. China; 4 Hubei Shizhen Laboratory, Wuhan, Hubei 430065 P.R. China

**Keywords:** Tongue squamous cell carcinoma, Capsaicin, Epithelial-mesenchymal transition, AMPK, HIF-1α, Claudin-1

## Abstract

**Background:**

Tongue squamous cell carcinoma (TSCC) is a highly aggressive malignancy with a strong propensity for early metastasis. Capsaicin (CAP), a major bioactive phytochemical isolated from *Capsicum* species, possesses significant anti-tumor activity. The present study aimed to elucidate the effects of CAP on epithelial-mesenchymal transition (EMT) and metastasis in TSCC, as well as to decipher the underlying molecular mechanisms.

**Results:**

Transcriptomic profiling of CAP-treated TSCC cells identified 5,904 DEGs, with GO enrichment in oxidative stress, metabolism and ECM pathways, and KEGG analysis highlighting the AMPK signaling pathway. Network pharmacology further revealed 72 shared targets between CAP and head and neck squamous cell carcinoma (HNSCC), predominantly enriched in the HIF-1 signaling pathway. In exploratory analyses of clinical samples (CPTAC database and 37 paired TSCC specimens), we observed an association of AMPK downregulation and HIF-1α upregulation with advanced tumor stage and poor differentiation. In vitro, CAP activated AMPK, suppressed HIF-1α, and consequently restored the epithelial junction protein Claudin-1, leading to the reversal of key EMT markers by upregulating E-cadherin and downregulating N-cadherin and Vimentin. Functionally, genetic manipulation established an AMPK/HIF-1α/Claudin-1 signaling axis: AMPK knockdown abrogated CAP-mediated effects, whereas HIF-1α overexpression mimicked EMT. Wound healing and Transwell assays verified that Claudin-1 deficiency rescued CAP-induced inhibition of cell migration. In vivo, CAP significantly reduced pulmonary metastatic nodules in nude mice in an AMPK-dependent manner, without inducing detectable hepatic or renal toxicity.

**Conclusions:**

Our study collectively delineates a novel mechanism wherein CAP constrains EMT and metastasis in TSCC through the AMPK/HIF-1α/Claudin-1 axis. These findings provide a mechanistic basis for understanding how CAP, a natural dietary constituent, interferes with TSCC progression.

**Supplementary Information:**

The online version contains supplementary material available at https://doi.org/10.1186/s13062-026-00938-6.

## Introduction

Tongue squamous cell carcinoma (TSCC) is the most common malignant tumor of oral cancer, characterized by high aggressiveness, local invasiveness, and a propensity for early cervical lymph node metastasis [1–3]. In recent years, its incidence has been increasing steadily and showing a trends in young-onset [4].

Capsaicin (CAP), a bioactive phytochemical derived from *Capsicum* species, exhibits relatively significant anti-tumor activity in vitro [5–9]. It demonstrates broad-spectrum inhibitory effects on migration and invasion of various malignancies, including breast cancer, melanoma, thyroid cancer, bladder cancer, small-cell lung cancer, and cholangiocarcinoma, while also inducing apoptosis and autophagy [10, 11].

It is well known that hypoxia develops when the tumor volume reaches 2 mm^3^, triggering tumor cells to produce hypoxia-inducible factor-1 (HIF-1) [12], which is a key transcription factor that can be activated under hypoxic conditions and consists of two subunits, α and β. HIF-1α is the specific oxygen-regulated subunit of HIF-1, which determines the activity of HIF-1 [13]. Under normoxic conditions, HIF-1α is rapidly degraded following synthesis, with a half-life of less than 5 min. When tissue oxygen tension declines, the expression of HIF-1α increases and accumulates in large quantities in the nucleus, forming a dimer with HIF-1β and becoming active, which can regulate the transcription of a battery of oxygen-sensitive genes (e.g., *VEGF*,* GLUT1*) to adapt to hypoxic stress [14].

Epithelial-mesenchymal transition (EMT) is a critical biological process that enables epithelial-derived tumor cells acquire invasive and migratory capabilities [15]. A hallmark of EMT in the initiation of tumor metastasis is the disruption of tight junction structures, which has directed research attention toward alterations in both tight junction molecules (notably Claudin family proteins) and adherens junction components like E-cadherin [16]. As the most extensively characterized and functionally pivotal member of the Claudin family, Claudin-1 is frequently downregulated or exhibits aberrant subcellular localization across diverse malignancies [17]. The Claudin-1 deficiency directly compromises intercellular tight junction integrity, disrupt cell-cell adhesion, and actively promotes EMT progression [18, 19]. In gastric cancer, the expression of Claudin-1 decreases with the increase of malignancy, during EMT, its expression is further downregulated. Notably, experimental overexpression of Claudin-1 exerts tumor-suppressive effects through a direct target of RUNX3 in gastric epithelial cells [20]. Our previous study also demonstrated that CAP combined with cisplatin inhibits TGF-β1-induced EMT and TSCC cells migration via the Claudin-1/AKT/mTOR signaling pathway [21].

In the present study, we further demonstrate that CAP activates AMPK, which in turn suppresses HIF-1α expression. This inhibition relieves HIF-1α-mediated transcriptional repression of Claudin-1, leading to the up-regulation of Claudin-1 protein expression, and consequent suppression of EMT and metastasis in TSCC cells.

## Methods

### Antibodies and reagents

CAP (HY-10448) and Capsazepine (CPZ, HY-15640) were purchased from MedChemExpress (USA, 126924). Primary antibodies included anti-E-cadherin (1:5000, 20874-1-AP), anti-N-cadherin (1:2000, 22018-1-AP), anti-Vimentin (1:1000, 10366-1-AP), anti-Claudin-1 (1:1000, 28674-1-AP) and anti-AMPK (1:1000, 10929-2-AP), all purchased from Proteintech. Anti-β-actin (1:5000, 8226) and anti-HIF-1α (1:1000, 179483) were purchased from Abcam. Anti-phosphorylated AMPKα (Thr172, 1:1000, BS5003), anti-TRPV1 (1:1000, BS80533) and peroxidase-conjugated anti-rabbit IgG secondary antibody (1:5000, BS22357) were obtained from Bioworld.

### Transcriptome analysis

Total RNA was extracted from collected CAP-treated and untreated TSCC cells. Transcriptome sequencing was performed by BGI (https://www.genomics.cn/). The corresponding library quality control protocol was selected based on product requirements. HISAT2 (v 2.2.1) was used to align the clean data to the reference genome. Bowtie2 (v 2.4.5) was employed to map the clean data to the reference gene set. RSEM (v 1.3.1) software was utilized for gene expression quantification, and the R package pheatmap (v 1.0.12) was used to generate the expression clustering heatmap of genes across different samples. DESeq2 (v 1.34.0) was applied for differential gene detection with the criteria of *Q-*value ≤ 0.05. To further explore the functions of genes associated with phenotypic changes, enrichment analysis of differentially expressed genes (DEGs) against the Gene Ontology (GO, http://www.geneontology.org/) and Kyoto Encyclopedia of Genes and Genomes (KEGG, https://www.kegg.jp/) databases was performed using Phyper. A threshold of *P*-value ≤ 0.05 was set, and those meeting this criterion were defined as significantly enriched among the candidate genes. Genes and gene sets with a False Discovery Rate (FDR) < 0.05 were considered statistically significant. To validate the differential expression results from DESeq2, edgeR was used for independent differential gene calling. For the statistical significance of functionally enriched gene sets, multivariate analysis of variance (MANOVA) was performed. FDR correction was applied to the results of both edgeR and MANOVA using the Benjamini-Hochberg method.

### Network pharmacology analysis

Key genes common to both CAP and TSCC were identified via network pharmacology analysis. First, the potential targeted genes of CAP were retrieved from two complementary databases: the Traditional Chinese Medicine Systems Pharmacology Database and Analysis Platform (TCMSP, https://www.tcmsp-e.com/) and the SwissTargetPrediction database (http://www.swisstargetprediction.ch/). Concurrently, disease-associated genes for head and neck squamous cell carcinoma (HNSCC) were collected from the GeneCards (https://www.genecards.org/) and Online Mendelian Inheritance in Man (OMIM, https://omim.org/) databases (with Relevance score ≥ 1.0). All retrieved genes were standardized to official Gene Symbols and duplicates removed to ensure data quality. The overlapping genes between the drug and disease were identified as potential therapeutic genes using the Venny (v 2.1.0) online tool (https://bioinfogp.cnb.csic.es/tools/venny/), resulting in 72 common genes. To elucidate the biological functions and pathways of these overlapping genes, GO enrichment analysis and KEGG pathway analysis were performed using the Metascape platform (https://metascape.org/).

### Clinical proteomic tumor analysis consortium (CPTAC) database analysis

TSCC is the most prevalent malignant neoplasm among HNSCC. Using the UALCAN platform (http://ualcan.path.uab.edu), we analyzed expression profiles of AMPK and HIF-1α in HNSCC from the CPTAC database (https://cptac-data-portal.georgetown.edu/).

### Tissue sample collection and immunohistochemical analysis

TSCC specimens (*n* = 37) and paired adjacent normal mucosal tissues (collected approximately 2 cm from tumor margins) were obtained from Peking University School of Stomatology. The study was approved by the Ethics Committee of the Peking University Health Science Center (Ethics Approval Number: IRB00001052-09087), and all participants signed informed consent forms. All specimens were fixed in 10% neutral buffered formalin, paraffin-embedded, and sectioned consecutively at 4 μm thickness. For immunohistochemistry (IHC), sections were deparaffinized and rehydrated through graded alcohols, followed by antigen retrieval in citrate buffer (0.01 mol/L, pH 6.0) using microwave heating for 5–10 min. Endogenous peroxidase activity was blocked with 3% H_2_O_2_ for 10 min at room temperature. After blocking with 5% horse serum for 20 min, primary antibodies were applied and incubated overnight at 4℃. The next day, sections were rewarmed and incubated with biotinylated secondary antibodies for 20 min at 37℃, followed by horseradish peroxidase-conjugated streptavidin for 30 min at 37℃. Color development was performed using 3,3’-diaminobenzidine substrate.

Immunostained sections were imaged using a LEICA 550 IW imaging system (Leica Microsystems, Germany). Positive staining was identified by brown-yellow granular deposits in cellular membranes and/or cytoplasm. Immunoreactive scores (IRS) were calculated according to Kaczorowski et al. [22]. as follows: IRS = mean staining intensity × percentage of positive cells. Both staining intensity and positive cell percentage were quantified using Image J software (NIH) following the method described by Leite et al. [23]. The final IRS for each specimen represented the average of five randomly selected fields.

### Cell culture and hypoxic treatment

The TSCC cell lines SCC9 (RRID: CVCL_1685), HN6 (RRID: CVCL_8129) and CAL27 (RRID: CVCL_1107) were procured from the American Type Culture Collection (ATCC). Cell authentication was confirmed by STR profiling. Mycoplasma contamination was tested using PCR detection (Mingzhoubio, China), and the latest test was negative in May 2025. Among them, the HN6 and CAL27 cell lines were cultured in DMEM high glucose medium (Biosharp Life Sciences, CN, BL304A) containing 10% fetal bovine serum (FBS) (Jitai Excellbio Biotechnology, FSP500) and 1% penicillin and streptomycin (Biosharp Life Sciences, BL505A). The SCC9 cell line was cultured in DMEM/F12 medium (Biosharp Life Sciences, BL305A) supplemented with 10% FBS, 2.5 mmol/L L-glutamine, 0.5 mmol/L sodium pyruvate, 1,200 mg/L NaHCO_3_, 15 mmol/L HEPES, 400 ng/mL hydrocortisone (Macklin Inc, H917755) and 1% penicillin and streptomycin. All cells were incubated at 37℃ in a humidified atmosphere containing 5% CO_2_ and passaged every 2 days.

Hypoxic conditions (1% O_2_, 5% CO_2_, and 94% N_2_) were established using a tri-gas incubator (HF100, Heal Force, Hong Kong, China). When cells reached 70% confluence, the medium was replaced with fresh complete medium before initiating hypoxic exposure. Cells were harvested at 0, 4, 8, and 12 h post-hypoxia induction for subsequent mRNA or protein extraction and analysis.

### Wound healing assay

Cells in logarithmic growth stage were seeded into 6-well plates. Following 24 h treatment with the predetermined optimal CAP concentration, a standardized scratch wound was created. Cell migration capacity was quantified by measuring residual scratch width at defined timepoints, with increased wound width correlating with impaired migratory ability.

### Transwell assay

Firstly, 500 µL of complete medium (10% FBS) was added to the lower chamber and incubated in the incubator for 30 min. After removing the conditioning medium, treated cells (6 × 10^4^ cells per well in serum-free medium) were seeded into the upper chamber. The lower chamber was then filled with complete medium containing the predetermined optimal concentration of CAP. Following 24 h incubation (37℃, 5% CO_2_), migrated cells were fixed with 4% paraformaldehyde for 15 min. Non-migrated cells on the upper membrane surface were removed by gentle swabbing with cotton-tipped applicators. Finally, migrated cells were stained with Giemsa solution for microscopic quantification.

### shRNA-mediated gene knockdown

To investigate the functional roles of AMPK, HIF-1α, and Claudin-1, we employed RNA interference using the following target-specific short hairpin RNA (shRNA) sequences:

AMPKα1: 

5′-GGAAGTTCTCAGCTGTCTTTA-3′.

HIF-1α: 

5′-GTGATGAAAGAATTACCGAAT-3′.

Claudin-1:

5′-GCAATAGAATCGTTCAAGA-3′.

The target sequence was cloned into the lentiviral plasmid vector pLenti-shRNA-GFP-Puro, after the successful vector construction was confirmed by PCR amplification and Sanger sequencing, the verified plasmids were amplified in DH5α competent cells with ampicillin selection, the high-titer viruses were produced by co-transfecting 293T cells with packaging plasmids, and the viral supernatants were concentrated using Amicon^®^ Ultra-15 ultrafiltration columns. Then, HN6 cells were transduced with lentivirus, puromycin (2 µg/mL) was used to select stably transfected populations, transduction efficiency was monitored by GFP fluorescence microscopy, and the knockdown efficiency was validated by Western blot analysis of target proteins.

### Construction and overexpression of a degradation-resistant HIF-1α (Pro564Ala) mutant

For overexpression of HIF-1α, the full-length coding sequence (CDS) of HIF-1α (*HIF1A*, GenBank: U22431.1, 3661 bp) was amplified. Given HIF-1α’s rapid degradation (< 5 min half-life) under normoxia via oxygen-dependent degradation domain (ODDD)-mediated ubiquitination, we introduced a Pro564Ala mutation in the ODDD region to stabilize the protein. This mutation prevents hydroxylation at Pro564, thereby blocking VHL-mediated ubiquitination and subsequent proteasomal degradation [24]. The HIF-1α (Pro564Ala) mutant was amplified using the following mutagenic primers (mutated codon in lowercase):

HIF1A-mut-Forward:

5′-TTGGAGATGTTAGCTgCCTATATCCCAATGG-3′,

HIF1A-mut-Reverse:

5′-CCATTGGGATATAGGcAGCTAACATCTCCAA-3′.

The PCR product was then cloned into the pEGFP-C1 mammalian expression vector between SacI and BamHI restriction sites. We designed the following cloning primers with appropriate restriction sites and protective bases: pEGFP-C1-HIF1A-SacI-Forward:5’-TCTCGAGCTCCCATGGAGGGCGCCGGCGGCGCGAACGAC-3’, pEGFP-C1-HIF1A-BamHI-Reverse:5’-CGGTGGATCCTCAGT TAACTTGATCCAAAGCT-3’. Following amplification with high-fidelity DNA polymerase, the PCR product and vector were double-digested with SacI and BamHI at 37℃ for 2 h. After agarose gel purification, the fragments were ligated using T4 DNA ligase at 16℃ overnight. The resulting plasmid, pEGFP-C1-HIF-1α (Pro564Ala), was verified by restriction analysis and Sanger sequencing. For stable expression, TSCC cells were transfected with the construct using Lipofectamine 3000 according to the manufacturer’s protocol. Stable transformants were selected with 800 µg/mL G418 for 2 weeks. Successful overexpression was confirmed by PCR amplification and Western blot analysis.

### Dual-Luciferase reporter assay

A 2000-bp DNA fragment encompassing the putative hypoxia response element (HRE) sequence (CGTG) within the Claudin-1 promoter was amplified from human genomic DNA by nested PCR. Primary PCR amplification was performed using the following primers:

481-CLDN1-Forward:

5′-CAGGCTAGGACACTTACCATAC-3′;

3506-CLDN1-Reverse:

5′-CTCCCTCAACTCGAACCACCAG-3′.

Followed by the secondary nested PCR was performed using the internal primers:

759-CLDN1-Forward: 

5′-TGAAGTAATCAGCCTCGCAC-3′;

3233-CLDN1-Reverse: 

5′-GTGCACTCACTGCTCAGATTC-3′.

The purified nested PCR product was cloned into the pProDuoLuci-Basic luciferase reporter vector using NotI/XhoI restriction sites. The cloning primers were designed with protective bases and enzyme sites: pProDuoLuci-Cln1-NotI-Forward: 5’- GCTTGCGGCCGCCAGTGGCACGATCTGGGCTCAC-3’, pProDuoLuci-Cln1-XhoI-Reverse: 5’- CCATCTCGAGGCAGAACCGCTGGGCGCCGCG-3’. Ligation products were transformed into E. coli DH5α, and positive clones were verified by Sanger sequencing.

For functional analysis, cells at 70% confluence in 96-well plates were co-transfected with the pProDuoLuci-Cln1 reporter plasmid and pEGFP-C1-HIF-1α (Pro564Ala) overexpression plasmid, and luciferase activity (Firefly/Renilla ratio) was measured at 48 h post-transfection using the Dual-Luciferase^®^ Reporter Assay System (Promega) under normoxia (21% O_2_), with relative luminescence units normalized to control transfections.

### Reverse transcription-quantitative PCR (RT-qPCR)

Total RNA was extracted from TSCC cells using the MolPure^®^ TRIeasy Plus Total RNA Kit (Yeasen Biotechnology. 19211ES60), and converted to cDNA with the PrimeScript™ RT Master Mix (Takara Biotechnology, RR036A). RT-qPCR analysis was performed with the SYBR Premix Ex Taq™ II (Takara Biotechnology, RR820A) in the CFX96 Real-Time PCR System (Bio-Rad, USA). The thermal cycling conditions for amplification were as follows: initial denaturation at 95 ℃ for 60 s, followed by 40 cycles at 95℃ for 15 s, 60℃ for 60 s, and 72℃ for 20 s. β-Actin was used as a reference gene for normalization. Primer sequences are listed below:

HIF-1α-Forward: 

5′-CAAAGACCCCAACGAGAAGC-3′,

HIF-1α-Reverse: 

5′-ATTTACTCCTATGCCGGCGA-3′,

Claudin-1-Forward: 

5′-ATTTACTCCTATGCCGGCGA-3′,

Claudin-1-Reverse: 

5′-ATTTACTCCTATGCCGGCGA-3′

β-Actin-Forward:

 5′-TGACGTGGACATCCGCAAAG-3′,

β-Actin-Reverse: 

5′-CTGGAAGGTGGACAGCGAGG-3′.

### Western blot

After treatment, total cellular proteins were extracted using RIPA buffer (Biosharp Life Sciences, BL504A) and quantified by BCA (Yeasen Biotechnology, 2020ES76) assay. Protein samples (30 µg) were mixed with 4×Loading buffer (Biosharp Life Sciences, BL529A), boiled at 95℃ for 8 min, and separated by 10% or 15% SDS-PAGE at 150 V for 60 min. Proteins were transferred to PVDF membranes (Millipore Corporation, IPVH00010) via wet transfer (100 V, 90 min). Membranes were blocked with 5% non-fat milk in TBST for 1 h at room temperature, followed by incubation with primary antibodies for 2 h at room temperature. Protein bands were visualized using ECL substrate (Biosharp Life Sciences, BL520B) and imaged with a Bio-Rad ChemiDoc™ MP Imaging System. Band intensities were quantified by Image J software (NIH, Bethesda, MD, USA).

### In vivo lung metastasis assay

Twenty male BALB/c nude mice (5-week-old) were procured from Zhuhai BesTest Co., Ltd, and housed under SPF conditions at the Experimental Animal Centre of Hubei University of Chinese Medicine (Ethics Approval Number: HUCMS24406389). After 1-week acclimatization, the mice were randomly divided into 4 groups (*n* = 5): Control (PBS), CAP (10 mg/kg, i.p., every 3 days), shAMPK, shAMPK + CAP. Lung metastatic models were established by tail vein injection of 1 × 10^6^ HN6 cells (in 200 µL PBS) or HN6 cells with stable knockdown of AMPK (1 × 10^6^). Intraperitoneal injections of CAP at a dosage of 10 mg/kg were administered from day 3 post-inoculation every 3 days. Body weight was monitored twice weekly. At 3 weeks, the mice were euthanized and their lungs, livers and kidneys were fixed in 4% paraformaldehyde, assessed through hematoxylin and eosin (HE) staining, and subsequently photographed under a microscope for further analysis.

### Statistical analysis

All quantitative data are presented as mean ± standard deviation (SD). Each experiment was conducted independently a minimum of 3 times (biological replicates). Statistical analyses were performed using GraphPad Prism 9.0. One-way ANOVA was utilized for single-variable comparisons, also followed by Tukey’s post hoc test. For multiple comparisons involving > 5 groups, Bonferroni correction was applied. For comparisons between two groups, an unpaired Student’s t-test was applied. A *P*-value of less than 0.05 was considered statistically significant.

## Results

### Transcriptomic combined with network pharmacology analysis predicts the targets and pathways of CAP in TSCC

**Fig. 1:**
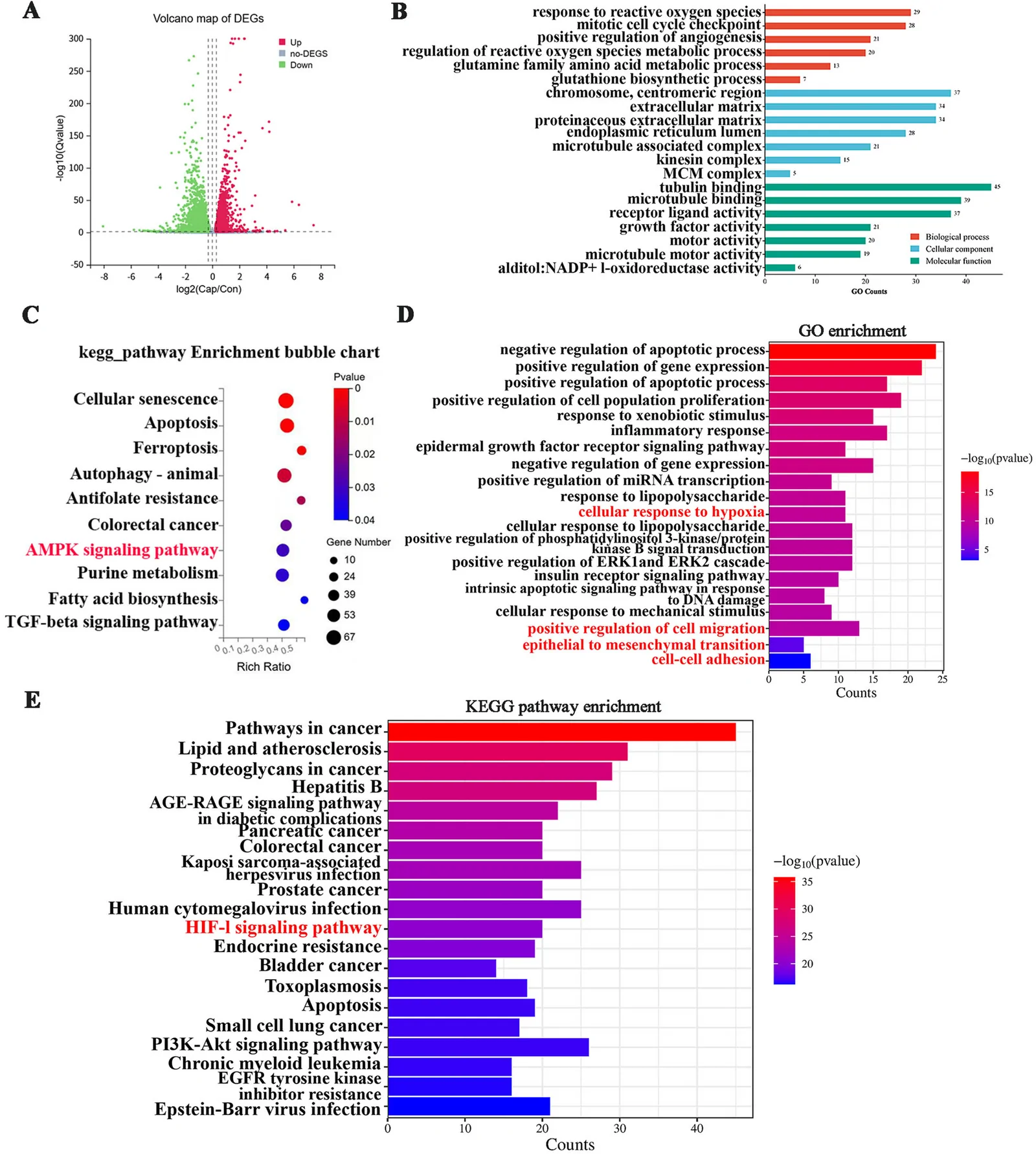
Transcriptomic and network pharmacology analysis predicts CAP target genes and pathways in TSCC. (**A**-**C**) DEGs between CAP-treated and untreated HN6 cells was identified by transcriptomic analysis, volcano plot of DEGs (**A**), categorical histogram on GO enrichment analysis (**B**) and bubble chart on KEGG enrichment analysis (**C**) of DEGs were displayed. (**D**, **E**) Key target genes and pathways of CAP-HNSCC were identified by network pharmacology analysis, categorical histogram showed the top 20 results by GO enrichment (**D**) and the top 20 pathways by KEGG enrichment analysis (**E**)

To elucidate the mechanism underlying the action of CAP in TSCC, we first performed transcriptomic analysis. By comparing RNA-seq data from CAP-treated and untreated TSCC cells, a total of 5,904 DEGs were identified, including 2,907 up-regulated and 2,997 down-regulated genes, which are visualized in the volcano plot (Fig. 1A). GO enrichment analysis revealed significant enrichment terms. Specifically, Biological Process (BP) terms were primarily related to oxidative stress and amino acid metabolism, Cellular Component (CC) terms were notably enriched for extracellular matrix (ECM) components (Fig. 1B). This suggests that CAP may regulate TSCC cell metabolism and interfere with ECM-associated structures or signaling pathways. Meanwhile, KEGG pathway analysis further identified significant enrichment of the AMPK signaling pathway, a central metabolic regulator (Fig. 1C).

To systematically predict the direct targets of CAP, a network pharmacology approach was subsequently employed. We retrieved 910 CAP-related genes from the TCMSP and SwissTargetPrediction databases, and 375 HNSCC-associated genes from the GeneCards and OMIM databases. Comparative analysis using Venny 2.1.0 revealed 72 overlapping genes, which represent putative therapeutic targets of CAP for TSCC. Subsequent enrichment analysis of these overlapping genes via Metascape highlighted significantly enriched pathways and biological processes. Specifically, GO-BP terms, including cellular response to hypoxia, EMT, and cell-cell adhesion, were significantly enriched (Fig. 1D). Furthermore, KEGG analysis identified significant enrichment in the HIF-1 signaling pathway (Fig. 1E).

Integrating these findings, AMPK (*PRKAA1/2*) and HIF-1α (*HIF1A*) were identified as candidate molecules for further investigation. We then examined their baseline expression in HNSCC using the GEPIA database. The results showed that *HIF1A* was significantly upregulated in tumor tissues, whereas *PRKAA2* exhibited a downward trend, although this change did not reach statistical significance. *PRKAA1* expression showed no appreciable difference between tumor and normal tissues (Fig. S1A). These bioinformatic observations, though derived from bulk tissue data and thus exploratory in nature, provided a working hypothesis that prompted us to further examine the protein expression of these candidates using our own clinical TSCC samples.

### Coordinated downregulation of AMPK and upregulation of HIF-1α associate with tumor progression in TSCC

Given that TSCC represents a major and clinically aggressive subtype within HNSCC, we subsequently sought to examine the protein expression of these candidate molecules in primary TSCC specimens. To this end, we first interrogated the CPTAC database (noting its inherent limitations as a bulk-tissue resource) and then performed immunohistochemistry staining on our own cohort of 37 paired TSCC clinical samples.

AMPK is a heterotrimeric complex composed of catalytic α subunits (α1 and α2), which are critical for its biological function [25]. To explore the relevance of these subunits in TSCC, we first analyzed the protein expression levels of AMPKα1 (*PRKAA1*), AMPKα2 (*PRKAA2*) and HIF-1α (*HIF1A*) using the CPTAC database. Compared with normal tissues, the expression of AMPKα1 remained relatively stable in tumor tissues, notably, it was downregulated in well-differentiated tumors (Grade 1) across different pathological stages (Fig. S1B-D). In contrast, AMPKα2 expression was significantly downregulated in HNSCC tissues (Fig. S1E), and this downregulation was consistently observed not only in advanced pathological stages (II-IV) but also in tumors of all differentiation grades (high, moderate, and poor) (Fig. S1F, G). Concurrently, HIF-1α protein levels were markedly elevated in tumors, with substantial increase observed in advanced-stage (stage IV) tumors and moderately differentiated tumors (Fig. S1H-J). These correlative data raise the hypothesis that an inverse expression pattern between AMPK and HIF-1α may be associated with tumor progression. We acknowledge that these observational analyses cannot establish causality and require functional experiments to verify potential regulatory relationships. Furthermore, as these data were derived from bulk tissue homogenates, we recognize that they do not distinguish tumor-cell-intrinsic expression from contributions of stromal or immune cells, therefore, they should be interpreted as hypothesis-generating rather than confirmatory.

**Fig. 2:**
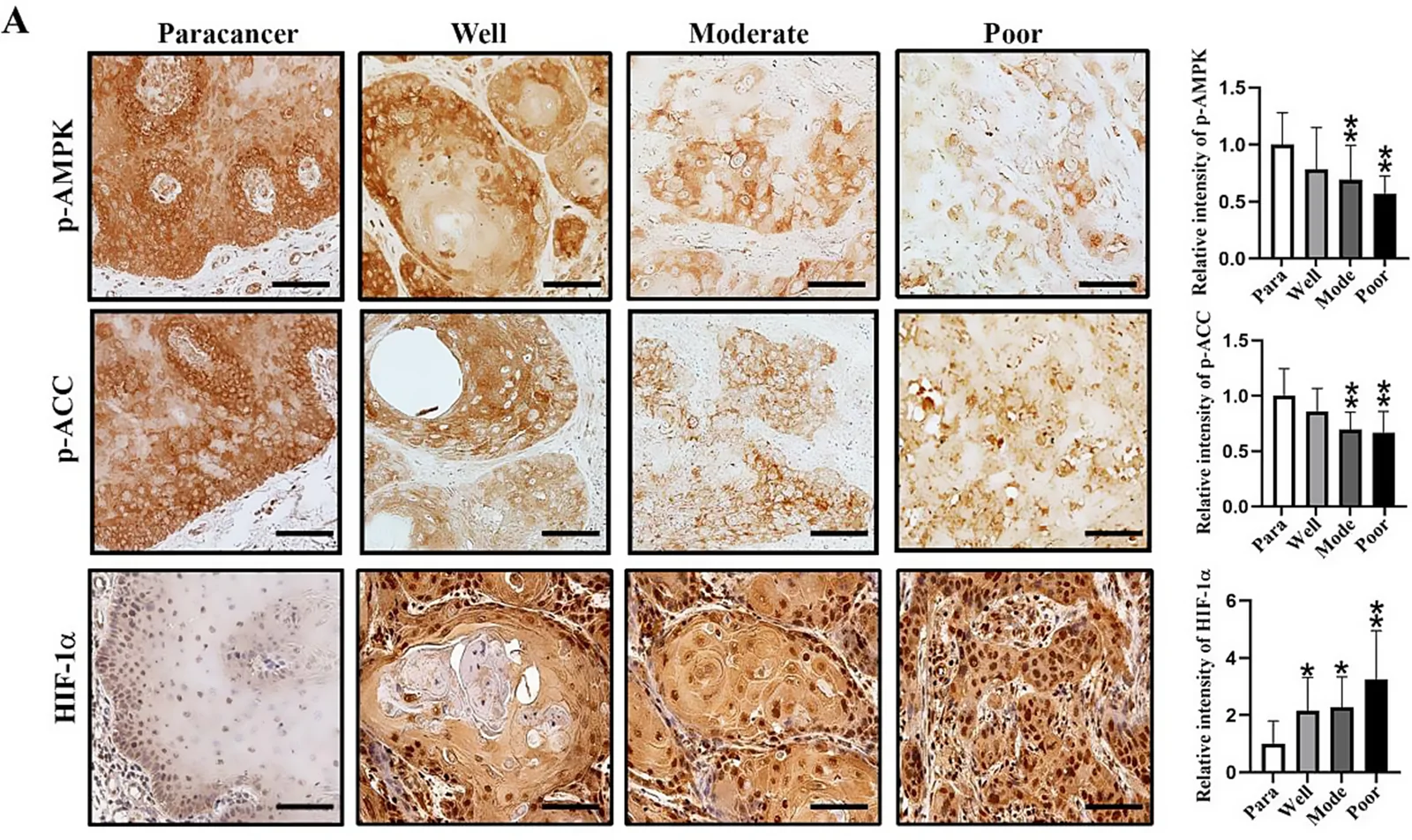
AMPK downregulation and HIF-1α upregulation correlate with TSCC progression. **(A)** IHC staining was performed to assess the expression of phosphorylated AMPK, phosphorylated ACC, and HIF-1α in TSCC tissues and adjacent non-cancerous tissues. Statistical significance is denoted by ^*^P < 0.05, ^**^P < 0.01 vs. Paracancer

Subsequently, in this exploratory study of 37 TSCC patients with TSCC, immunohistochemistry was performed to examine the expression of p-AMPK, p-ACC and HIF-1α (Fig. 2A), and we compared the protein expression levels of p-AMPK, p-ACC, and HIF-1α between adjacent normal tissues and tumors stratified by pathological stage and differentiation grade (Table S1, S2). p-AMPK expression was significantly lower in stage II tumors (mean difference = − 0.35, 95% CI: − 0.64 to − 0.06) and in moderately (mean difference = − 0.27, 95% CI: − 0.51 to − 0.03) and poorly differentiated tumors (mean difference = − 0.43, 95% CI: − 0.79 to − 0.08). p-ACC showed similar significant reductions in stage II (mean difference = − 0.26, 95% CI: − 0.48 to − 0.05), stage III (mean difference = − 0.37, 95% CI: − 0.63 to − 0.12), and in moderately (mean difference = − 0.30, 95% CI: − 0.48 to − 0.12) and poorly differentiated tumors (mean difference = − 0.33, 95% CI: − 0.60 to − 0.07). HIF-1α was significantly elevated only in moderately (mean difference = 1.16, 95% CI: 0.18–2.15) and poorly differentiated tumors (mean difference = 2.23, 95% CI: 0.64–3.83), with no significant differences by pathological stage. Due to the small sample size, these findings are preliminary and require validation in larger independent cohorts. Nevertheless, the observed trends-specifically, decreased p-AMPK/p-ACC in higher-grade tumors and elevated HIF-1α in poorly differentiated tumors-are biologically plausible and consistent with the development of a characteristic hypoxic microenvironment in advanced TSCC. Collectively, downregulation of AMPK and upregulation of HIF-1α were identified in TSCC patient samples, suggesting that there may be an interaction between metabolic dysfunction and hypoxia adaptation in TSCC cells.

### CAP modulates the AMPK/HIF-1α/Claudin-1 axis to suppress EMT and migration in TSCC cells

**Fig. 3:**
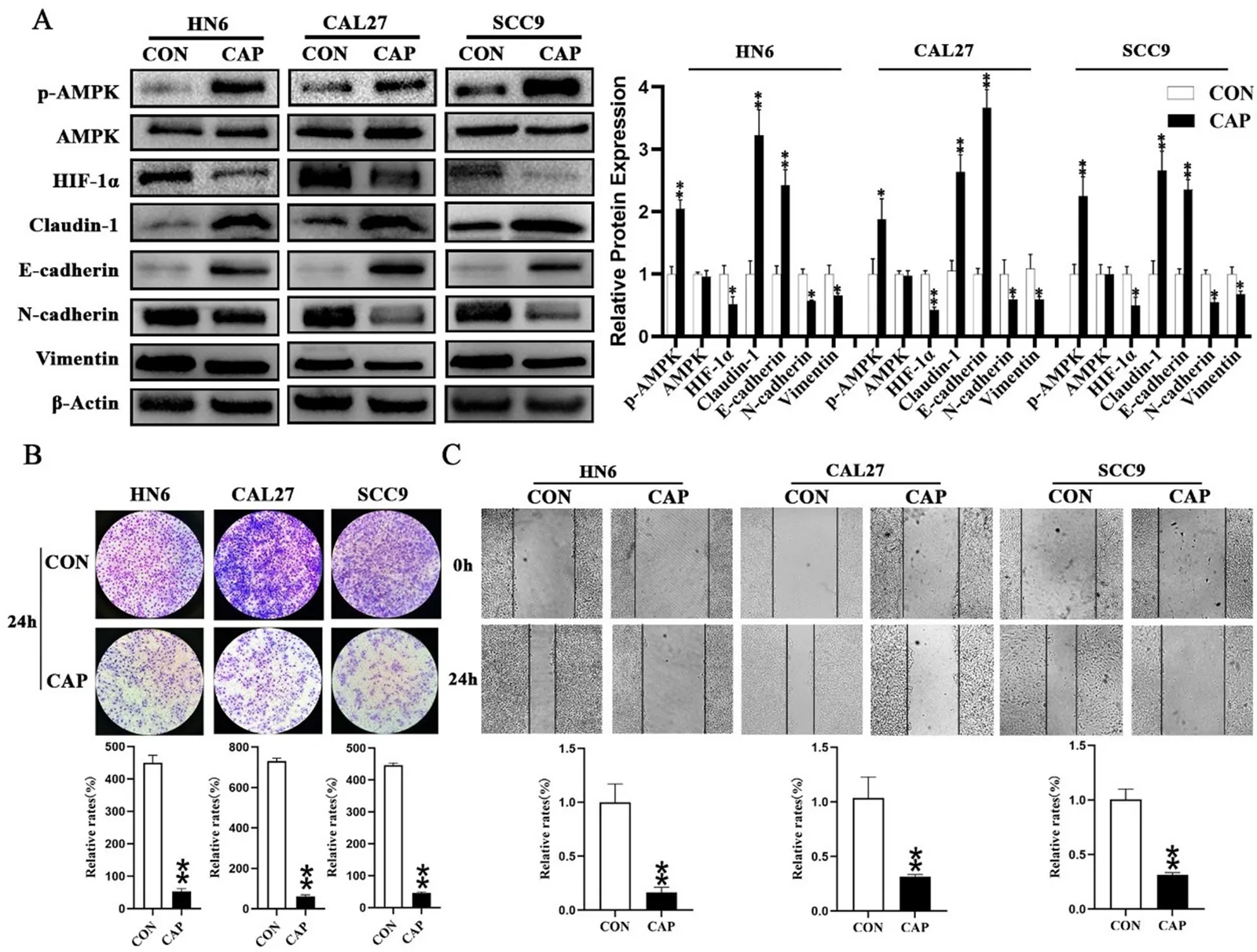
CAP modulates the AMPK/HIF-1α/Claudin-1 axis to suppress EMT and migration in TSCC cells. (**A**) HN6, CAL27, and SCC9 cells were treated with CAP (150 µM) for 24 h, and the expression of the AMPK/HIF-1α/Claudin-1 signaling axis and EMT-related proteins (E-cadherin, N-cadherin, Vimentin) was analyzed by western blot. (**B**,** C**) Cell migration was assessed using wound healing and Transwell assays. After 24 h of CAP treatment, the closure distance and number of migrated cells were photographed (×20 magnification) and measured. Data are presented as mean ± SD of 3 independent experiments. Statistical significance is denoted by ^*^P < 0.05, ^**^P < 0.01 vs. CON. CON: control, CAP: capsaicin

In our previous study, CAP exhibited significantly higher cytotoxicity against tumor cells than against normal oral keratinocytes [26]. To further delineate the mechanism underlying its anti-migration effects, we examined its impact on the key components of the AMPK/HIF-1α/Claudin-1 axis under the established optimal treatment conditions (150 µM, 24 h) in HN6, CAL27, and SCC9 cell lines [21]. Western blot analysis demonstrated that CAP treatment potently activated AMPK signaling, as indicated by increased phosphorylation of AMPK, while concurrently suppressing HIF-1α protein expression. Consistent with its inhibitory effect on EMT, CAP markedly upregulated epithelial junction proteins Claudin-1 and E-cadherin, while downregulating the key mesenchymal markers N-cadherin and Vimentin (Fig. 3A). Functionally, CAP significantly impaired the migratory capacity of TSCC cells, as evidenced by Transwell and wound healing assays (Fig. 3B, C).

To further investigate whether the observed AMPK activation by CAP is mediated through the transient receptor potential vanilloid 1 (TRPV1) channel, we employed the selective TRPV1 antagonist CPZ in subsequent mechanistic experiments. After verifying the appropriate CPZ concentration gradient in HN6 cells, we assessed its effect on CAP-induced signaling. Western blot analysis revealed that CPZ pretreatment effectively blocked CAP-triggered TRPV1 upregulation, however, it failed to abrogate CAP-stimulated phosphorylation of AMPK (Fig. S2A, B).

Taken together, these findings demonstrate that CAP suppresses EMT and cell migration in TSCC by targeting the AMPK/HIF-1α/Claudin-1 signaling axis, an effect that occurs independently of TRPV1.

### AMPK mediates CAP-induced regulation of the HIF-1α/Claudin-1 axis and suppression of EMT in TSCC

**Fig. 4:**
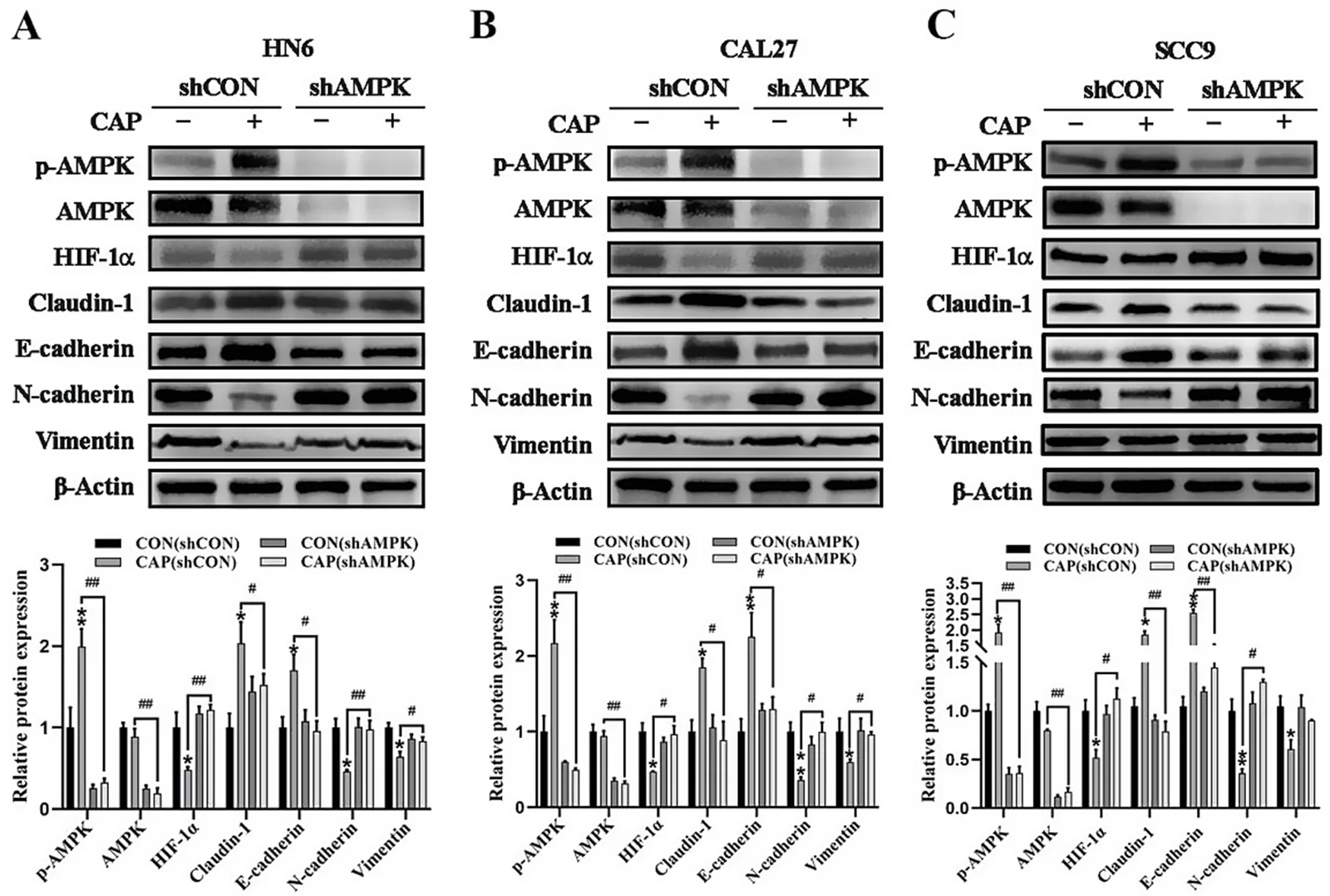
AMPK mediates CAP-induced regulation of the HIF-1α/Claudin-1 axis and suppression of EMT in TSCC. (**A-C**) The expression of AMPK was knockdown by shRNA interference in HN6, CAL27 and SCC9 cells, and cells were cultured with CAP (150 µM) for 24 h. The expression of the AMPK/HIF-1α/Claudin-1 signaling axis and EMT-related proteins (E-cadherin, N-cadherin, Vimentin) was analyzed by western blot. Data are presented as mean ± SD of 3 independent experiments. Statistical significance is denoted by ^*^P < 0.05, ^**^P < 0.01 vs. CON (shCON), and ^#^*P* < 0.05, ^##^*P* < 0.01 for CAP (shAMPK) vs. CAP (shCON). CON: control, CAP: capsaicin

To investigate the mechanistic role of AMPK in the regulatory effects of CAP, we generated AMPK-knockdown TSCC cell lines through shRNA-mediated interference. Strikingly, AMPK depletion significantly attenuated CAP-induced downregulation of HIF-1α and upregulation of Claudin-1. Furthermore, AMPK-deficient cells exhibited marked resistance to CAP-mediated EMT suppression. Compared to CAP-treated control cells, AMPK-knockdown cells showed reduced E-cadherin and elevated N-cadherin and Vimentin levels even in the presence of CAP (Fig. 4A, B). To exclude potential off-target effects, we validated the key findings using another independent shRNA of AMPK gene in HN6 cell. The results obtained with alternative shRNA were consistent with those from the initial knockdown experiments (Fig. S3). Collectively, these findings demonstrate that AMPK acts as a central mediator that is required for CAP to concurrently inhibit HIF-1α, restore Claudin-1, and suppress the EMT program in TSCC cells.

### HIF-1α mediates CAP-induced Claudin-1 upregulation and EMT suppression in TSCC

**Fig. 5:**
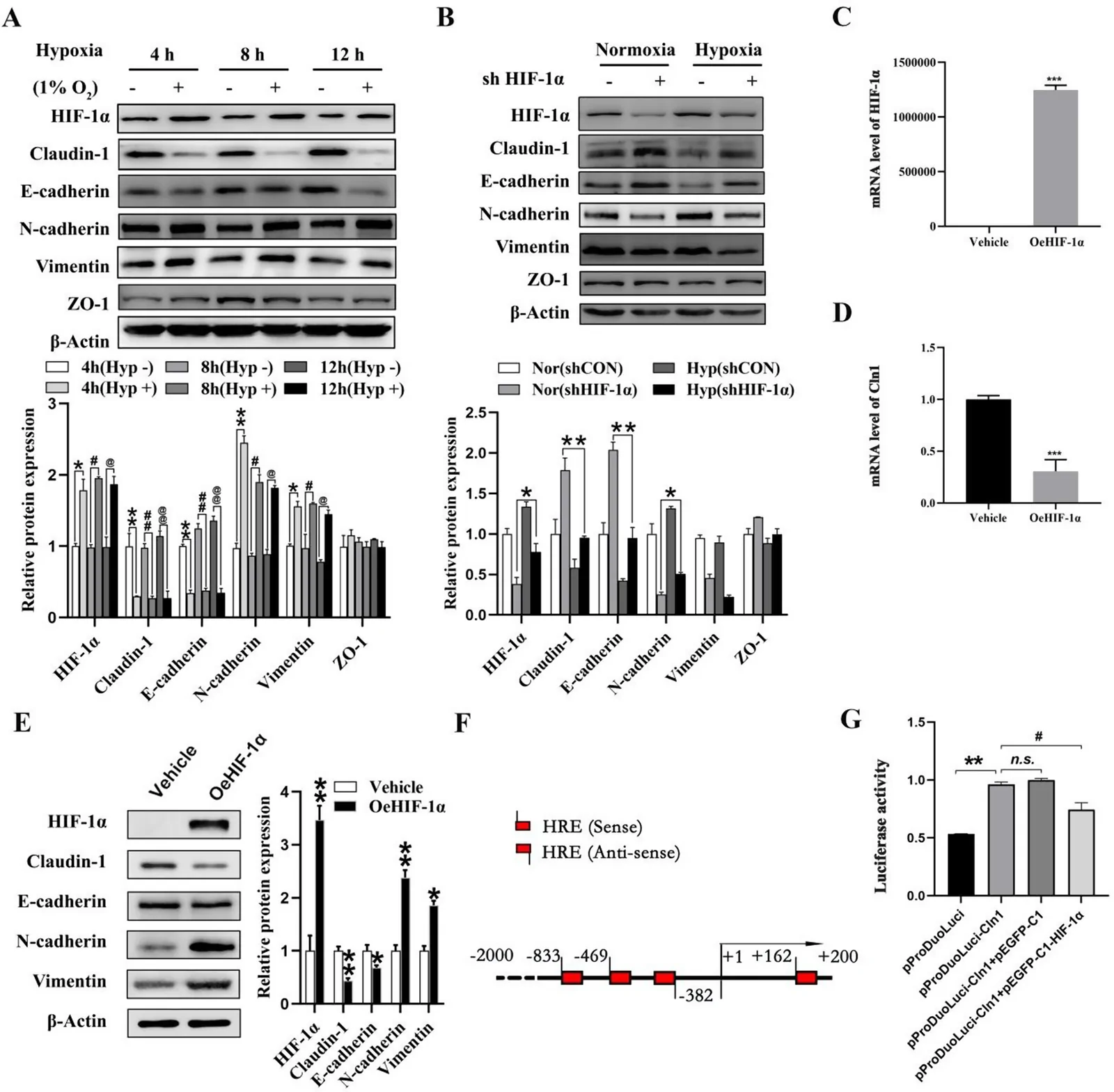
HIF-1α regulates Claudin-1 by binding to its promoter region and influences EMT. (**A**) HN6 cells were cultured with hypoxic treatment for 4, 8, and 12 h, and the expression of HIF-1α, Claudin-1, ZO-1 and EMT-related proteins was analyzed by western blot. Statistical significance is denoted by ^*^P < 0.05, ^**^P < 0.01 vs. 4 h (Hyp-), ^*#*^*P* < 0.05, ^*##*^*P* < 0.01 vs. 8 h (Hyp-), ^*@*^*P* < 0.05, ^*@@*^*P* < 0.01 vs. 12 h (Hyp**-**). (**B**) The expression of HIF-1α, Claudin-1, ZO-1 and EMT-related proteins in HN6 cells with stable HIF-1α- knockdown was analyzed by western blot under both normoxia and hypoxia conditions (8 h); Statistical significance is denoted by ^*^P < 0.05, ^**^P < 0.01 for Nor (shHIF-1α) vs. Hyp (shHIF-1α). (**C**,** D**) Quantitative analysis was performed to assess the relative expression of HIF-1α and Claudin-1 mRNA in HN6 cells transfected with HIF-1α overexpression vector compared to control cells. (**E**) The expression of HIF-1α, Claudin-1 and EMT-related proteins in HN6 cells transfected with HIF-1α overexpression vector was analyzed by western blot; Statistical significance is denoted by ^*^P < 0.05, ^**^P < 0.01 vs. Vehicle. (**F**) The sites of HREs in Claudin-1 promoter region (-2000 to + 200 bp). (**G**) HN6 cells were co-transfected with the Claudin-1 promoter-luciferase reporter and HIF-1α expression vector, and luciferase activity was normalized to the control. Statistical significance is denoted by ^**^P < 0.01 vs. pProDuoLuci, ^*#*^*P* < 0.05 pProDuoLuci-Cln1 vs. pProDuoLuci-Cln1 + pEGFP-C1-HIF-1α. Data are presented as mean ± SD of 3 independent experiments. Hyp: Hypoxia, Nor: Normoxia

To delineate the functional role of HIF-1α in the regulatory effects of CAP, HN6 cells were exposed to hypoxia (1% O_2_) for 4, 8 and 12 h. Hypoxic treatment rapidly induced HIF-1α protein accumulation, concomitant with suppression of Claudin-1 and E-cadherin, as well as the induction of Vimentin and N-cadherin expression. Notably, the tight junction protein ZO-1 remained unchanged (Fig. 5A). Genetic ablation of HIF-1α abrogated these hypoxia-driven molecular alterations under both normoxic and hypoxic conditions (Fig. 5B), thus establishing HIF-1α as a master regulator of Claudin-1 and EMT markers.

To directly probe the HIF-1α/Claudin-1 axis, we engineered a stable HIF-1α-overexpressing TSCC cell model using the pEGFP-C1-HIF-1α plasmid. RT-qPCR and immunoblotting confirmed successful overexpression of HIF-1α at mRNA and protein levels respectively (Fig. 5C, E). Strikingly, HIF-1α overexpression suppressed Claudin-1 expression and induced EMT, as evidenced by downregulation of E-cadherin and concomitant upregulation of N-cadherin and Vimentin (Fig. 5D, E). Finally, we further treated HIF-1α-overexpressed cells with CAP. The results revealed that CAP reversed the pro-EMT effect induced by HIF-1α overexpression (Fig. S4).

Taken together, these results establish HIF-1α as an upstream regulator of Claudin-1 and EMT markers in TSCC. Furthermore, the observation that CAP reverses HIF-1α-driven EMT indicates that CAP exerts its anti-EMT effects, at least in part, through modulation of HIF-1α and subsequent restoration of Claudin-1 expression in TSCC.

### HIF-1α represses Claudin-1 transcription by binding to its promoter region

As a master hypoxia-responsive transcription factor, HIF-1α regulates gene expression by binding to conserved HREs containing the core (A/G) CGTG motif [27]. Bioinformatic analysis of the Claudin-1 promoter region (− 2000 to + 200 bp) identified four putative HREs (Fig. 5F). To functionally validate this regulation, we cloned the Claudin-1 promoter fragment into a luciferase reporter vector (pProDuoLuci-Cln1), and co-transfected it with either HIF-1α-overexpressing plasmid (pEGFP-C1-HIF-1α) or empty vector control (pEGFP-C1). Dual-luciferase assays demonstrated that HIF-1α overexpression significantly suppressed Claudin-1 promoter-driven luciferase activity compared to the empty vector control (Fig. 5G). These results demonstrate that HIF-1α transcriptionally represses Claudin-1 expression, likely through binding to HREs within its promoter region, thereby contributing to EMT progression in TSCC cells.

### Claudin-1 mediates CAP-induced suppression of EMT and cell migration in TSCC

**Fig. 6:**
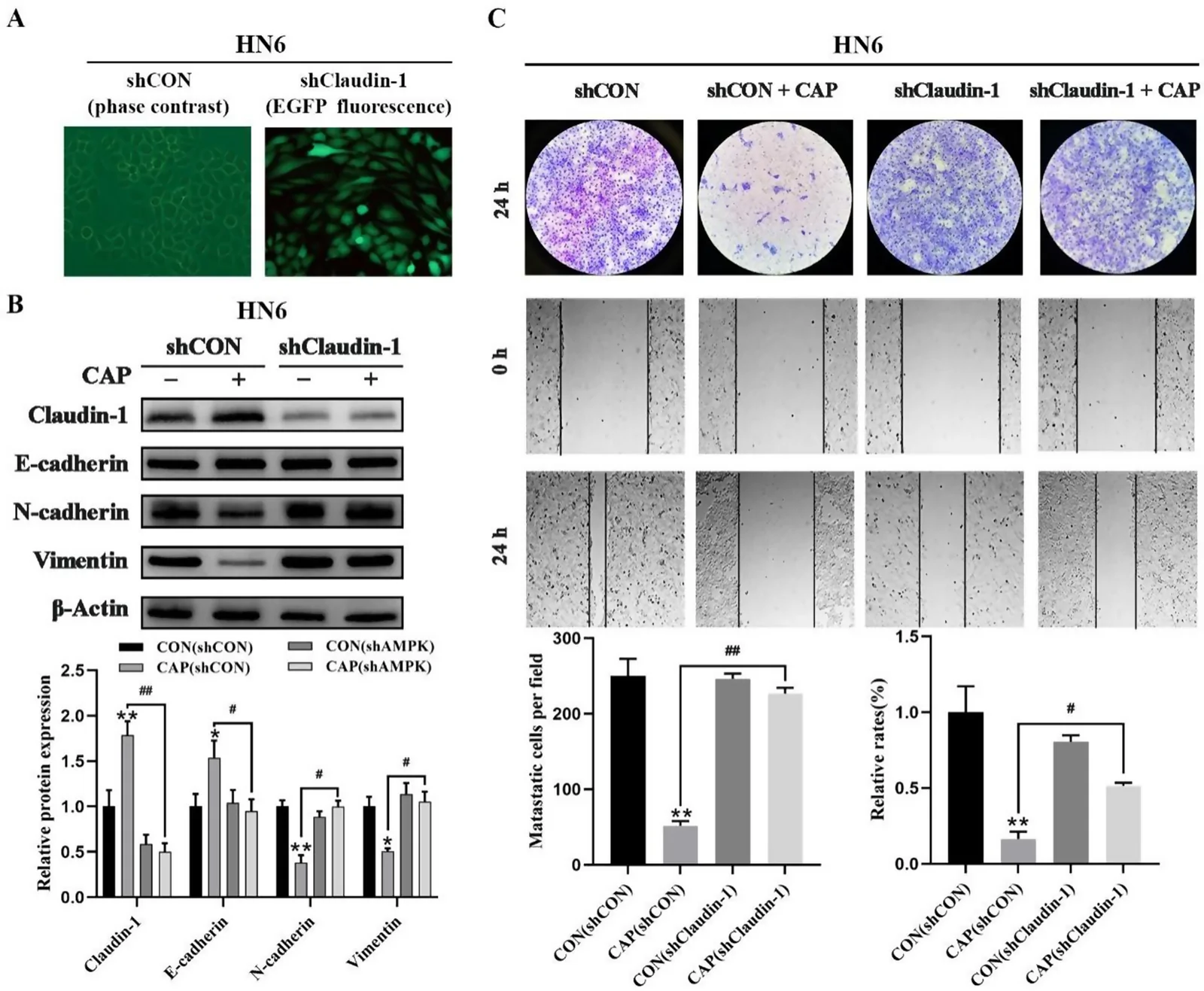
Claudin-1 mediates CAP-induced EMT suppression and migration inhibition in TSCC cells.** (A)** Fluorescence microscopy was utilized to examine the cellular morphological alterations subsequent to the knockdown of Claudin-1. **(B)** Cells were cultured with CAP (150 µM) for 24 h, and the expression of Claudin-1 and EMT-related proteins (E-cadherin, N-cadherin, Vimentin) was analyzed by western blot. **(C)** Cell migration was assessed using Transwell and wound healing assays. After 24 h of CAP treatment, the number of migrated cells and closure distance were photographed (×20 magnification) and measured. Data are presented as mean ± SD of 3 independent experiments. Statistical significance is denoted by ^**^P < 0.01 vs. CON (shCON), and ^#^*P* < 0.05, ^##^*P* < 0.01 for CAP (shClaudin-1) vs. CAP (shCON)

To define the functional role of Claudin-1 in CAP-mediated suppression of EMT and cell migration, we generated Claudin-1-deficient TSCC cells through shRNA interference (Fig. 6A, B). Morphological analysis demonstrated that Claudin-1 knockdown induced a partial mesenchymal transition, characterized by the acquisition of a spindle-like morphology in a subset of cells, contrasting with the epithelial polygonal morphology observed in controls (Fig. 6A). At the molecular level, Claudin-1 depletion markedly attenuated the regulatory effects of CAP. Specifically, it compromised CAP-induced upregulation of E-cadherin and blunted the downregulation of the mesenchymal markers N-cadherin and Vimentin (Fig. 6B). Functionally, Claudin-1 deficiency significantly rescued the inhibitory effect of CAP on cell migration, as shown in Transwell and wound healing assays (Fig. 6C). The specificity of the knockdown phenotypes was confirmed using another independent shRNA targeting Claudin-1 in HN6 cells, which yielded concordant results (Fig. S5). Collectively, these data identify Claudin-1 as a critical downstream effector through which CAP exerts its inhibitory effects on both EMT and migratory capacity in TSCC cells.

### CAP suppresses TSCC metastasis via AMPK activation in vivo without systemic toxicity

**Fig. 7:**
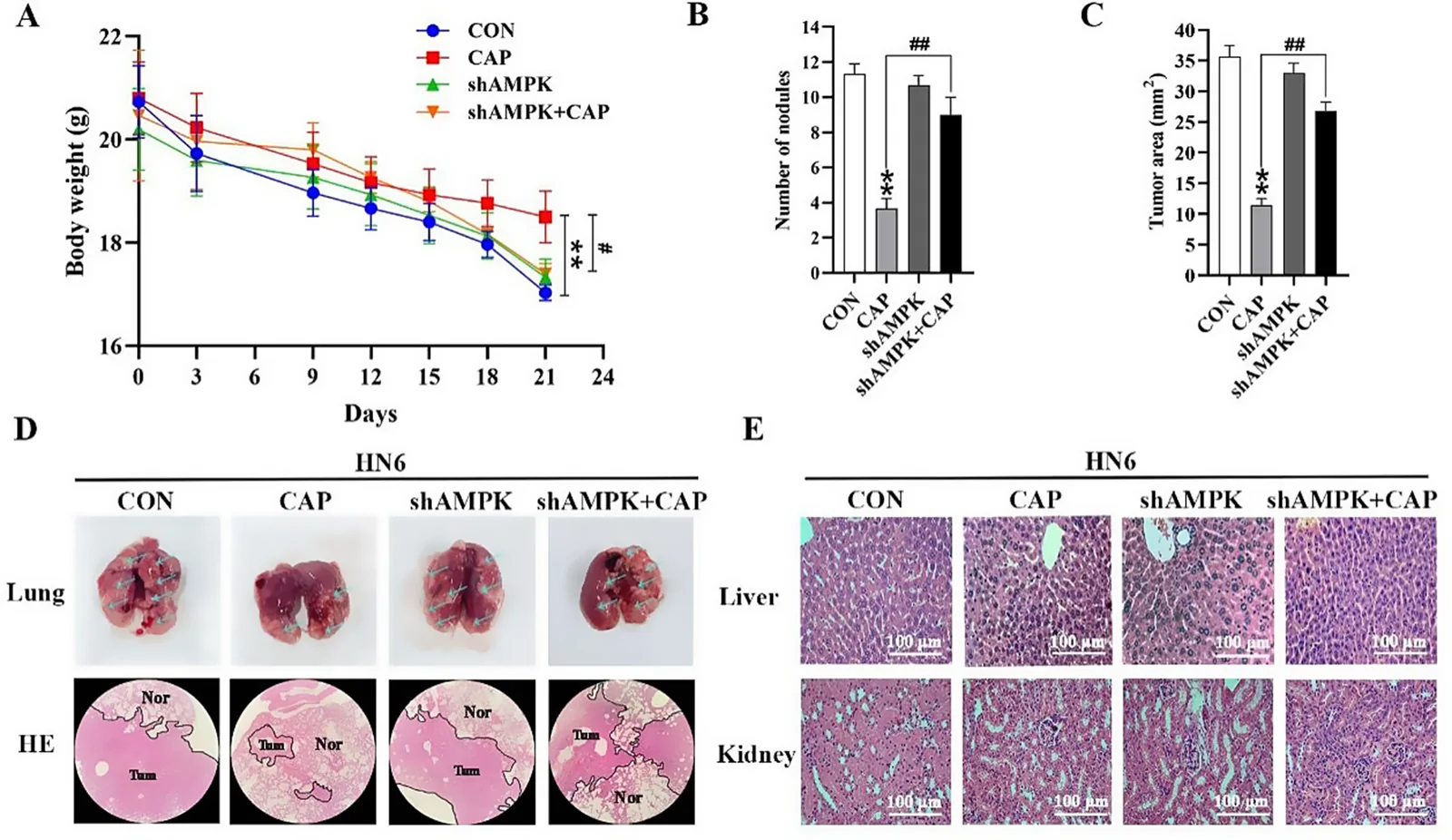
CAP suppresses TSCC metastasis via AMPK activation in vivo without systemic toxicity. **(A)** Changes in the body weight of mice over 21 days. **(B)** Number of metastatic nodules in the lung of nude mice. **(C)** Tumor area of lung metastases in nude mice. **(D)** The lungs of the mice were harvested, photographed, and tumor metastasis was assessed via HE staining (×100). **(E)** The tissue sections of mice livers and kidneys were stained with HE and drug toxicity was evaluated by histopathological morphology (×200). Statistical significance is denoted by ^**^P < 0.01 vs. CON, and ^##^*P* < 0.01 for shAMPK + CAP vs. CAP. Nor: Normal, Tum: Tumor

To evaluate the anti-metastatic efficacy of CAP in vivo, we established a lung metastasis model by tail vein injection of HN6 cells (wild-type or AMPK-knockdown) into nude mice. In mice bearing control HN6 cells, CAP treatment significantly attenuated body weight loss in mice (Fig. 7A). Meanwhile, CAP also markedly inhibited tumor metastasis, leading to an approximately 70% reduction in the number of lung metastatic nodules (Fig. 6B, D) and a 65% decrease in tumor area (Fig. 7C, D). Collectively, these results demonstrate that CAP treatment improves the overall health status and alleviates tumor burden. In stark contrast, AMPK deficiency completely abolished the anti-metastatic effect of CAP. Mice bearing AMPK-knockdown tumors showed no significant reduction in metastatic nodules or tumor area upon CAP treatment, and their body weight loss mirrored that of the untreated group (Fig. 7A-D). This unequivocally establishes that CAP’s efficacy is AMPK-dependent. Importantly, CAP treatment elicited no detectable systemic toxicity. Histopathological examination demonstrated intact hepatic and renal architecture in CAP-treated mice, with no signs of necrosis, inflammatory infiltration or fibrosis (Fig. 7E). In conclusion, these in vivo findings demonstrate that CAP exerts potent and AMPK-dependent anti-metastatic activity against TSCC, while exhibiting a favorable safety profile, supporting its therapeutic potential.

## Discussion

Both normal and neoplastic cells rely on precise oxygen homeostasis to sustain physiological functions. The rapid proliferation of tumor cells frequently exceeds vascular perfusion capacity, thereby generating the characteristic hypoxic microenvironment that is a hallmark of solid tumors [28, 29]. In turn, this hypoxic stress triggers elaborate adaptive cellular responses governed by transcriptional and proteomic reprogramming [30–32]. A central mediator of such adaptive response is HIF-1α, a master transcriptional regulator that orchestrates cellular hypoxic adaptation by modulating energy metabolism, metastatic potential, angiogenesis and chemoresistance [33, 34]. However, in TSCC, the clinical significance of HIF-1α-mediated hypoxic response and its potential link to AMPK signaling remain incompletely understood, which prompted our present investigation. Our clinical analyses revealed prominent overexpression of HIF-1α in TSCC tissues, which was strongly and positively correlated with advanced clinical stage and high pathological grade. Furthermore, immunohistochemical examination of 37 paired TSCC clinical specimens demonstrated a progressive reduction in AMPK phosphorylation (Thr172) with increasing tumor grade. Conversely, HIF-1α expression displayed an inverse trend, with maximal levels detected in poorly differentiated TSCC tumors. Taken together, these clinical observations indicate an inverse functional relationship between AMPK activity and HIF-1α expression during TSCC progression. This intriguing correlation thus warrants further mechanistic investigations to delineate the precise molecular crosstalk between these two signaling mediators.

To address this knowledge gap, we performed in vitro assays to explore the regulatory crosstalk among AMPK, HIF-1α, and CAP in TSCC cells. CAP is derived from chili peppers, a kind of *Solanaceae* plant with food-medicine homology. Accumulating evidence has demonstrated that CAP exerts beneficial effects against inflammation, obesity and pain [35–37]. Moreover, preclinical studies have indicated that CAP impedes malignant progression in multiple tumor types [5]. In the present study, we found that CAP treatment markedly inhibited HIF-1α expression while inducing AMPK phosphorylation, notably, these molecular changes were concomitant with potent suppression of EMT in TSCC cells. Importantly, these results are consistent with previous reports demonstrating CAP-mediated AMPK activation in prostate cancer and hepatocellular carcinoma [38, 39], while further expanding this research field by uncovering a novel tumor-suppressive role of CAP in TSCC through distinct downstream molecular mechanisms. Although CAP has been shown to inhibit tumor progression and EMT in multiple malignancies, including renal cell carcinoma, nasopharyngeal carcinoma and osteosarcoma [40–42], our study is the first to demonstrate that CAP elicits anti-EMT effects in TSCC specifically by modulating the AMPK/HIF-1α signaling axis. To further characterize the underlying mechanism, we identified Claudin-1 as a key downstream effector in this pathway, linking CAP-mediated metabolic regulation to its anti-metastatic effects in TSCC cells and thereby establishing a complete molecular cascade through which CAP constrains TSCC progression.

Our genetic manipulation studies provide compelling evidence for a central role of AMPK in mediating the anti-migration effects of CAP. AMPK knockdown significantly abrogated CAP-mediated regulation of the HIF-1α/Claudin-1 axis and the subsequent suppression of EMT in TSCC cells, Complementary in vivo experiments further confirmed that CAP exerts its anti-metastatic activity in an AMPK-dependent manner. Accumulating evidence has demonstrated that HIF-1α accumulation promotes angiogenesis and EMT in lung cancer [43] and drives metastatic progression in hepatocellular carcinoma [44]. In line with this findings, our gain- and loss-of-function assays verified that HIF-1α silencing upregulated Claudin-1 expression and reduced the levels of mesenchymal markers, whereas HIF-1α overexpression induced the opposite phenotypic effects. As a canonical hypoxia-responsive transcription factor, HIF-1α executes its transcriptional regulatory function by binding to HREs in the promoter regions of its target genes. The conserved core motif of HREs represents the minimal cis-regulatory element essential for HIF-1α binding [27]. We therefore analyzed the Claudin-1 promoter region and identified 4 HRE motifs within this regulatory region. Dual-luciferase reporter assays subsequently provided direct functional evidence that HIF-1α transcriptionally represses Claudin-1 expression through binding to its promoter region, thereby establishing a novel mechanistic connection between hypoxia signaling and tight junction regulation during malignant progression of TSCC. This HIF-1α/Claudin-1 regulatory axis represents a previously unrecognized molecular pathway through which CAP exerts its anti-EMT effects in TSCC.

The present study demonstrates that CAP treatment significantly upregulates Claudin-1 expression in TSCC cells. As a critical component of tight junctions and a well-characterized modulator of EMT, Claudin-1 exerts complex, context-dependent biological roles in tumor progression [45, 46]. Accumulating studies have reported that Claudin-1 elicits a bidirectional regulatory effect on tumorigenesis, mediating both pro-tumorigenic and anti-tumorigenic properties in a cancer-type specific manner [47, 48]. For example, numerous studies have documented aberrant Claudin-1 overexpression in aggressive malignancies, including hepatocellular carcinoma [49], colorectal carcinoma [50], and breast cancer [51], where its upregulation correlates with poor prognosis and promotes tumor invasion. In contrast, a growing body of evidence supports prominent tumor-suppressive roles of Claudin-1 in prostate cancer [52], triple-negative breast cancer [53] and colorectal cancer [54]. In this study, CAP-induced upregulation of Claudin-1 clearly and inhibited cell migration. Specifically, genetic depletion of Claudin-1 substantially attenuated CAP’s capacity to suppress EMT-related markers (abrogating CAP-induced E-cadherin upregulation and rescuing N-cadherin expression) and cell migration (evidenced by an increased wound closure rate compared with CAP-treated control cells). Collectively, these consistent findings from molecular and functional assays validate Claudin-1 as a crucial mediator of CAP’s anti-metastatic activity in TSCC, highlighting its potential as a therapeutic target for this malignancy.

**Fig. 8:**
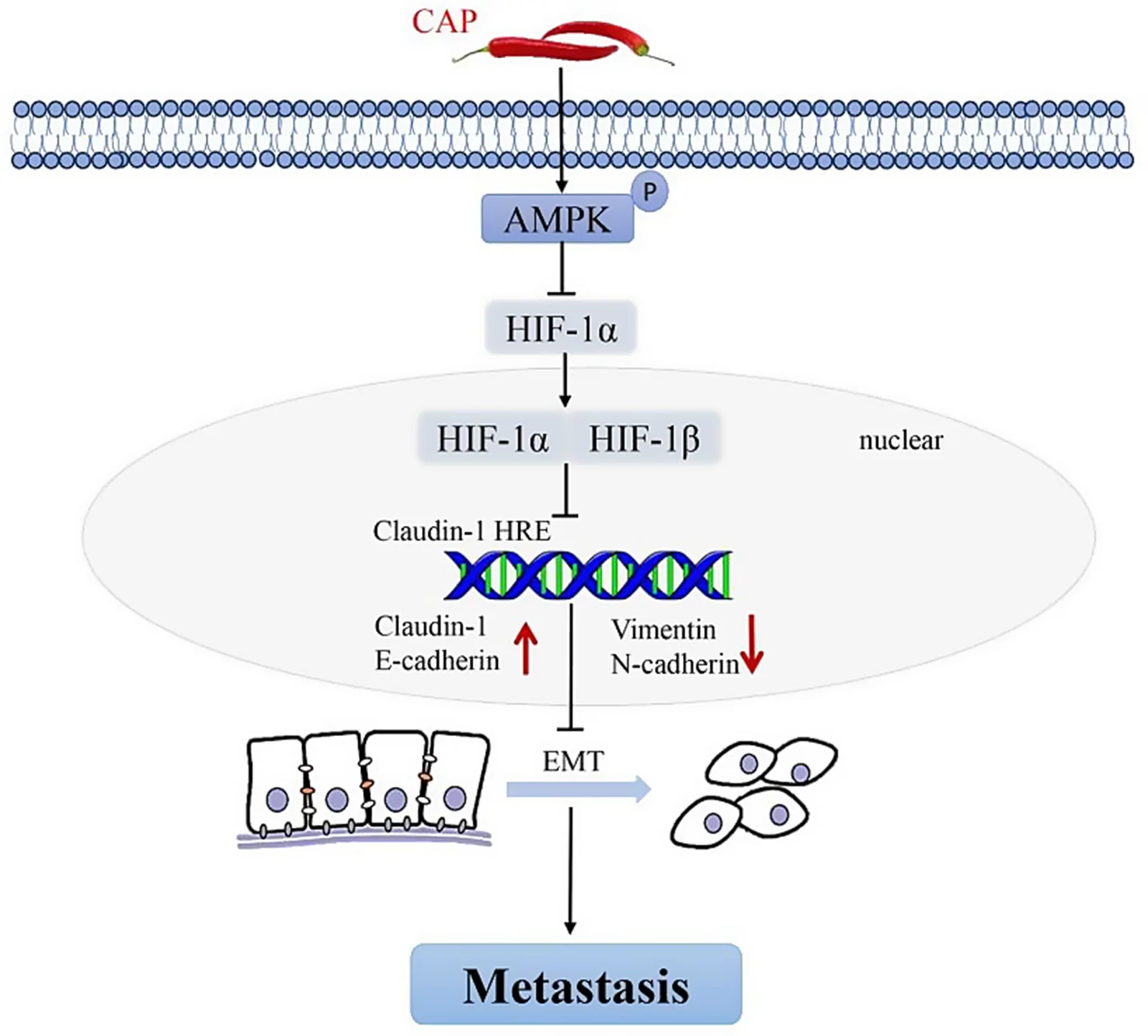
Diagram illustrates that CAP inhibits EMT and migration in TSCC through the AMPK/HIF-1α/Claudin-1 pathway. CAP activates AMPK, which in turn suppresses HIF-1α expression. This inhibition relieves HIF-1α-mediated transcriptional repression of Claudin-1, leading to the upregulation of the epithelial markers Claudin-1 and E-cadherin, and the downregulation of the mesenchymal markers Vimentin and N-cadherin, and consequent suppression of EMT and metastasis of TSCC cells

In conclusion, our study elucidates a novel mechanistic pathway through which CAP exerts anti-migration activity in TSCC cell lines in vitro. Specifically, we demonstrate that CAP activates the AMPK signaling, which in turn downregulates HIF-1α and upregulates Claudin-1, ultimately inhibiting EMT and metastatic progression (Fig. 8), but the mechanism by which CAP activates AMPK was not determined. These findings position CAP as a promising therapeutic candidate for TSCC treatment, particularly given its demonstrated efficacy and favorable toxicity profile in our in vivo models. Importantly, CAP’s multi-targeted modulation of the AMPK/HIF-1α/Claudin-1 axis suggests potential advantages over single-target therapies, which may mitigate the risk of drug resistance emergence.

Several limitations should be acknowledged. The sample size is small (*n* = 37), and subgroup sizes by differentiation grade or pathological stage are even smaller, which limits statistical power and the precision of effect estimates (as reflected by wide 95% confidence intervals in Tables S1 and S2). Multiple comparisons were corrected using the Bonferroni method, but the risk of type II errors remains high in underpowered subgroups. Therefore, all findings are exploratory and hypothesis-generating. Independent validation in a larger cohort is required before any clinical or biomarker-related claims can be made. Furthermore, the public database analyses (TCGA/GTEx/CPTAC) included in this study are derived from bulk tissues and do not account for tumor purity or cellular heterogeneity, accordingly, these data were used only as exploratory leads and were placed in the Supplementary Information to avoid overinterpretation. In addition, our pharmacological data suggest that CAP activates AMPK through a TRPV1-independent pathway, however, the precise upstream mechanism awaits further investigation.

In summary, our study collectively delineates a novel mechanism wherein CAP constrains EMT and metastasis in TSCC through the AMPK/HIF-1α/Claudin-1 axis. These findings not only provide preclinical evidence supporting CAP’s anti-metastatic properties but also preliminarily elucidate the molecular mechanism by which this natural bioactive constituent suppresses TSCC progression, highlighting its potential as a promising therapeutic candidate for this malignancy.

## Supplementary Information

Below is the link to the electronic supplementary material.

Supplementary Material 1

Supplementary Material 2

Supplementary Material 3

Supplementary Material 4

Supplementary Material 5

Supplementary Material 6

Supplementary Material 7

Supplementary Material 8

Supplementary Material 9

Supplementary Material 10

Supplementary Material 11

Supplementary Material 12

Supplementary Material 13

Supplementary Material 14

## Data Availability

The data supporting the findings of this study are available upon request from the corresponding author.

## Abbreviations

TSCC: Tongue squamous cell carcinoma
CAP: Capsaicin
EMT: Epithelial-mesenchymal transition
HIF-1α: Hypoxia-inducible factor-1α
TCMSP: Traditional Chinese Medicine Systems Pharmacology
GO: Gene ontology
KEGG: Kyoto encyclopedia of genes and genomes
CPTAC: Clinical proteomic tumor analysis consortium
HNSCC: Head and neck squamous cell carcinoma
IRS: Immunoreactive scores
ATCC: American type culture collection
FBS: Fetal bovine serum
shRNA: short hairpin RNA
CDS: Coding sequence
ODDD: Oxygen-dependent degradation domain
HRE: Hypoxia response element
RT-qPCR: Reverse transcription-quantitative PCR
AMPK: AMP-activated protein kinase

## References

[1] Matsuo K, Akiba J, Kusukawa J, Yano H. Squamous cell carcinoma of the tongue: subtypes and morphological features affecting prognosis. Am J Physiol Cell Physiol. 2022;323(6):C1611–23. 10.1152/ajpcell.00098.2022 36252129

[2] da Silva Souto AC, Vieira Heimlich F, de Lima L. Epidemiology of tongue squamous cell carcinoma: A retrospective cohort study. Oral Dis. 2023;29(2):402–10. 10.1111/odi.13897 33964106

[3] Luo D, Shi F, Wang S, Yang J, Zhou R. Tongue squamous cell carcinoma resists hyperthermia treatment by promoting Id-1 expression mediated EMT. Bull Cancer. 2022;109(9):886–94. 10.1016/j.bulcan.2022.05.004 35788271

[4] Magnes T, Egle A, Greil R, Melchardt T. Update on squamous cell carcinoma of the head and neck: ASCO annual meeting 2017. Memo. 2017;10(4):220–3. 10.1007/s12254-017-0358-9 PMC5725515 29250200

[5] Merritt JC, Richbart SD, Moles EG, et al. Anti-cancer activity of sustained release capsaicin formulations. Pharmacol Ther. 2022;238:108177. 10.1016/j.pharmthera.2022.108177 PMC9510151 35351463

[6] Cheng P, Wu J, Zong G, Wang F, et al. Capsaicin shapes gut microbiota and pre-metastatic niche to facilitate cancer metastasis to liver. Pharmacol Res. 2023;188:106643. 10.1016/j.phrs.2022.106643 36608780

[7] Deng R, Yu S, Ruan X, Liu H, Zong G, et al. Capsaicin orchestrates metastasis in gastric cancer via modulating expression of TRPV1 channels and driving gut microbiota disorder. Cell Commun Signal. 2023;21(1):364. 10.1186/s12964-023-01265-3 PMC10734064 38129926

[8] Brown KC, Sugrue AM, Conley KB. Anti-cancer activity of capsaicin and its analogs in gynecological cancers. Adv Cancer Res. 2024;164:241–81. 10.1016/bs.acr.2024.05.005 39306367

[9] Arivalagan N, Ramakrishnan A, Sindya J, et al. Capsaicin promotes apoptosis and inhibits cell migration via the tumor necrosis factor-Alpha (TNFα) and nuclear factor Kappa B (NFκB) signaling pathway in oral cancer cells. Cureus. 2024;16(9):e69839. 10.7759/cureus.69839 PMC11492975 39435241

[10] Friedman JR, Richbart SD, Merritt JC. Capsaicinoids: multiple effects on angiogenesis, invasion and metastasis in human cancers. Biomed Pharmacother. 2019;118:109317. 10.1016/j.biopha.2019.109317 PMC6759410 31404777

[11] Chu H, Li M, Wang X. Capsaicin induces apoptosis and autophagy in human melanoma cells. Oncol Lett. 2019;17(6):4827–34. 10.3892/ol.2019.10206 PMC6507355 31186689

[12] Xiong Q, Liu B, Ding M. Hypoxia and cancer related pathology. Cancer Lett. 2020;486:1–7. 10.1016/j.canlet.2020.05.002 32439418

[13] Ke Q, Costa M. Hypoxia-inducible factor-1 (HIF-1). Mol Pharmacol. 2006;70(5):1469–80. 10.1124/mol.106.027029 16887934

[14] Korbecki J, Simińska D, Gąssowska-Dobrowolska M. Chronic and cycling hypoxia: drivers of cancer chronic inflammation through HIF-1 and NF-κB activation: a review of the molecular mechanisms. Int J Mol Sci. 2021;22(19):10701. 10.3390/ijms221910701 PMC8509318 34639040

[15] Lüönd F, Sugiyama N, Bill R, Bornes L. Distinct contributions of partial and full EMT to breast cancer malignancy. Dev Cell. 2021;56(23):3203–e322111. 10.1016/j.devcel.2021.11.006 34847378

[16] Zhang Y, Weinberg RA. Epithelial-to-mesenchymal transition in cancer: complexity and opportunities. Front Med. 2018;12(4):361–73. 10.1007/s11684-018-0656-6 PMC6186394 30043221

[17] Nishii N, Oshima T, Li M, Eda H, et al. Lubiprostone induces claudin-1 and protects intestinal barrier function. Pharmacology. 2020;105(1–2):102–8. 10.1159/000503054 31536982

[18] Xia Y, Cao H, Zheng J, Chen L. Claudin-1 mediated tight junction dysfunction as a contributor to atopic march. Front Immunol. 2022;13:927465. 10.3389/fimmu.2022.927465 PMC9277052 35844593

[19] Ouban A. Claudin-1 role in colon cancer. An update and a review. Histol Histopathol. 2018;33(10):1013–9. 10.14670/HH-11-980 29504115

[20] Chang TL, Ito K, Ko TK, et al. Claudin-1 has tumor suppressive activity and is a direct target of RUNX3 in gastric epithelial cells. Gastroenterology. 2010;138(1):255–65. 10.1053/j.gastro.2009.08.044 19706291

[21] Li Z, Zhao Q, Liu X, Zhou X, Wang Y, et al. Capsaicin combined with cisplatin inhibits TGF-β1-induced EMT and TSCC cells migration via the Claudin-1/PI3K/AKT/mTOR signaling pathway. Cancer Cell Int. 2024;24(1):300. 10.1186/s12935-024-03485-0 PMC11360848 39198820

[22] Kaczorowski M, Biecek P, Donizy P, et al. SMAD7 is a novel independent predictor of survival in patients with cutaneous melanoma. Transl Res. 2019;204:72–81. 10.1016/j.trsl.2018.09.002 30342000

[23] Leite AF, Bernardo VG, Buexm LA, Fonseca EC, et al. Immunoexpression of cleaved caspase-3 shows lower apoptotic area indices in lip carcinomas than in intraoral cancer. J Appl Oral Sci. 2016;24(4):359–65. 10.1590/1678-775720160156 PMC4990365 27556207

[24] Ivan M, Kondo K, Yang H, Kim W, et al. HIFalpha targeted for VHL-mediated destruction by proline hydroxylation: implications for O2 sensing. Science. 2001;292(5516):464–8. 10.1126/science.1059817 11292862

[25] Trefts E, Shaw RJ. AMPK: restoring metabolic homeostasis over space and time. Mol Cell. 2021;81(18):3677–90. 10.1016/j.molcel.2021.08.015 PMC8549486 34547233

[26] Zhou XY, Zhao QW, Li Z, et al. Capsaicin exerts synergistic pro-apoptotic effects with cisplatin in TSCC through the calpain pathway via TRPV1. J Cancer. 2024;15(15):4801–17. 10.7150/jca.98075 PMC11310884 39132151

[27] Cowman SJ, Koh MY. Revisiting the HIF switch in the tumor and its immune microenvironment. Trends Cancer. 2022;8(1):28–42. 10.1016/j.trecan.2021.10.004 PMC8702465 34743924

[28] Goyette MA, Elkholi IE, Apcher C. Targeting Axl favors an antitumorigenic microenvironment that enhances immunotherapy responses by decreasing Hif-1α levels. Proc Natl Acad Sci USA. 2021;118(29):e2023868118. 10.1073/pnas.2023868118 PMC8307381 34266948

[29] Davis L, Recktenwald M, Hutt E. Targeting HIF-2α in the tumor microenvironment: redefining the role of HIF-2α for solid cancer therapy. Cancers (Basel). 2022;14(5):1259. 10.3390/cancers14051259 PMC8909461 35267567

[30] Darie CC, Hukovic A, Maynard VD, Neagu AN. Roles of oxygen in the tumorigenesis, progression, and treatment of breast cancer. Med Gas Res. 2026;16(1):41–5. 10.4103/mgr.MEDGASRES-D-25-00023 PMC12318570 40580187

[31] Tao J, Yang G, Zhou W, et al. Targeting hypoxic tumor microenvironment in pancreatic cancer. J Hematol Oncol. 2021;14(1):14. 10.1186/s13045-020-01030-w PMC7805044 33436044

[32] Sasidharan Nair V, Saleh R, Toor SM, et al. Metabolic reprogramming of T regulatory cells in the hypoxic tumor microenvironment. Cancer Immunol Immunother. 2021;70(8):2103–21. 10.1007/s00262-020-02842-y PMC8289790 33532902

[33] Zhang J, Ouyang F, Gao A. ESM1 enhances fatty acid synthesis and vascular mimicry in ovarian cancer by utilizing the PKM2-dependent warburg effect within the hypoxic tumor microenvironment. Mol Cancer. 2024;23(1):94. 10.1186/s12943-024-02009-8 PMC11077861 38720298

[34] Bai R, Li Y, Jian L, Yang Y, et al. The hypoxia-driven crosstalk between tumor and tumor-associated macrophages: mechanisms and clinical treatment strategies. Mol Cancer. 2022;21(1):177. 10.1186/s12943-022-01645-2 PMC9454207 36071472

[35] Thongin S, Den-Udom T, Uppakara K, et al. Beneficial effects of capsaicin and dihydrocapsaicin on endothelial inflammation, nitric oxide production and antioxidant activity. Biomed Pharmacother. 2022;154:113521. 10.1016/j.biopha.2022.113521 36007275

[36] Zsiborás C, Mátics R, Hegyi P, et al. Capsaicin and capsiate could be appropriate agents for treatment of obesity: A meta-analysis of human studies. Crit Rev Food Sci Nutr. 2018;58(9):1419–27. 10.1080/10408398.2016.1262324 28001433

[37] Arora V, Campbell JN, Chung MK. Fight fire with fire: Neurobiology of capsaicin-induced analgesia for chronic pain. Pharmacol Ther. 2021;220:107743. 10.1016/j.pharmthera.2020.107743 PMC7969397 33181192

[38] Sánchez BG, Bort A, Mora-Rodríguez JM, et al. The natural chemotherapeutic capsaicin activates AMPK through LKB1 kinase and TRPV1 receptors in prostate cancer cells. Pharmaceutics. 2022;14(2):329. 10.3390/pharmaceutics14020329 PMC8880011 35214061

[39] Bort A, Sánchez BG, Mateos-Gómez PA, Díaz-Laviada I, et al. Capsaicin targets lipogenesis in HepG2 cells through AMPK activation, AKT inhibition and PPARs regulation. Int J Mol Sci. 2019;20(7):1660. 10.3390/ijms20071660 PMC6480012 30987128

[40] Que T, Ren B, Fan Y. Capsaicin inhibits the migration, invasion and EMT of renal cancer cells by inducing AMPK/mTOR-mediated autophagy. Chem Biol Interact. 2022;366:110043. 10.1016/j.cbi.2022.110043 36044967

[41] Chiang C, Zhang M, Wang D, et al. Therapeutic potential of targeting MKK3-p38 axis with capsaicin for nasopharyngeal carcinoma. Theranostics. 2020;10(17):7906–20. 10.7150/thno.45191 PMC7359099 32685028

[42] Chen ZY, Huang HH, Li QC, et al. Capsaicin reduces cancer stemness and inhibits metastasis by downregulating SOX2 and EZH2 in osteosarcoma. Am J Chin Med. 2023;51(4):1041–66. 10.1142/S0192415X23500489 37120706

[43] Zheng Y, Ji H, Yi W, Chen Z. PRMT5 facilitates angiogenesis and EMT via HIF-1α/VEGFR/Akt signaling axis in lung cancer. Aging. 2023;15(13):6163–78. 10.18632/aging.204826 PMC10373979 37400960

[44] Xu H, Zhao J, Li J, Zhu Z, et al. Cancer associated fibroblast-derived CCL5 promotes hepatocellular carcinoma metastasis through activating HIF1α/ZEB1 axis. Cell Death Dis. 2022;13(5):478. 10.1038/s41419-022-04935-1 PMC9119971 35589690

[45] Blanchard AA, Ma X, Wang N. Claudin 1 is highly upregulated by PKC in MCF7 human breast cancer cells and correlates positively with PKCε in patient biopsies. Transl Oncol. 2019;12(3):561–75. 10.1016/j.tranon.2018.12.011 PMC6349319 30658316

[46] Takasawa K, Takasawa A, Akimoto T. Regulatory roles of claudin-1 in cell adhesion and microvilli formation. Biochem Biophys Res Commun. 2021;565:36–42. 10.1016/j.bbrc.2021.05.070 34090208

[47] Bhat AA, Syed N, Therachiyil L, et al. Claudin-1, a double-edged sword in cancer. Int J Mol Sci. 2020;21(2):569. 10.3390/ijms21020569 PMC7013445 31952355

[48] Fatima I, Uppada JP, Chhonker YS. Identification and characterization of a first-generation inhibitor of claudin-1 in colon cancer progression and metastasis. Biomed Pharmacother. 2023;159:114255. 10.1016/j.biopha.2023.114255 PMC10824272 36696800

[49] Stebbing J, Filipović A, Giamas G. Claudin-1 as a promoter of EMT in hepatocellular carcinoma. Oncogene. 2013;32(41):4871–2. 10.1038/onc.2012.591 23318416

[50] Bhat AA, Ahmad R, Uppada SB, et al. Claudin-1 promotes TNF-α-induced epithelial-mesenchymal transition and migration in colorectal adenocarcinoma cells. Exp Cell Res. 2016;349(1):119–27. 10.1016/j.yexcr.2016.10.005 PMC6166648 27742576

[51] Mattern J, Roghi CS, Hurtz M, et al. ADAM15 mediates upregulation of Claudin-1 expression in breast cancer cells. Sci Rep. 2019;9(1):12540. 10.1038/s41598-019-49021-3 PMC6715704 31467400

[52] Zhu L, Tang N, Hang H. Loss of Claudin-1 incurred by DNMT aberration promotes pancreatic cancer progression. Cancer Lett. 2024;586:216611. 10.1016/j.canlet.2024.216611 38309617

[53] Geoffroy M, Kleinclauss A, Kuntz S, et al. Claudin 1 inhibits cell migration and increases intercellular adhesion in triple-negative breast cancer cell line. Mol Biol Rep. 2020;47(10):7643–53. 10.1007/s11033-020-05835-3 32979166

[54] Kim NY, Pyo JS, Kang DW, Yoo SM. Loss of claudin-1 expression induces epithelial-mesenchymal transition through nuclear factor-κB activation in colorectal cancer. Pathol Res Pract. 2019;215(3):580–5. 10.1016/j.prp.2019.01.015 30683478

