# Supplementary material for: Capsaicin suppresses metastasis in tongue squamous cell carcinoma by reversing epithelial-mesenchymal transition via the AMPK/HIF-1α/Claudin-1 axis
Source: Biol Direct. 2026 Aug 11;21:232. doi: 10.1186/s13062-026-00938-6 (PMC13746642; doi:10.1186/s13062-026-00938-6)
Supplement: Supplementary file 1 [file 13062_2026_938_MOESM12_ESM.docx]

**Fig. S1.** Analysis of HIF-1α and AMPK expression based on public databases **(A)** The mRNA levels of *HIF1A, PRKAA1*, and *PRKAA2* were analyzed in tumor and normal tissues using the GEPIA database. **(B-J)** Changes in AMPKα1 (*PRKAA1*), AMPKα2 (*PRKAA2*), and HIF-1α (*HIF1A*) protein expression in HNSCC were analyzed using the CPTAC database.Statistical significance is denoted by ^*^*P* < 0.05 *vs*. Normal group.

**Fig.S2.** Pharmacological inhibition of TRPV1 fails to block CAP-triggered AMPK activation. **(A)** HN6 cells were treated with gradient concentrations of CPZ (0, 5, 10, 15 μM) to determine the optimal intervention condition. **(B)** HN6 and CAL27 cells were subjected to CAP, CPZ, or combined CAP and CPZ treatment. TRPV1 and p-AMPK protein expression were analyzed by western blot. Shown are representative blots from two independent biological replicates.

**Fig. S3.** Effects of AMPK knockdown on CAP-induced expression of HIF-1α, Claudin-1, and EMT-related proteins in TSCC cells. The HN6 cell were transfected with shAMPK (5'-GCTTGATGCACACATGAATGC-3') or shCON (control) and then treated with CAP or left untreated (control). Protein expression levels of p-AMPK, total AMPK, HIF-1α, Claudin-1, E-cadherin, N-cadherin, and Vimentin were analyzed by western blot. β-Actin served as a loading control. The results demonstrate that AMPK knockdown abrogates CAP-induced HIF-1α suppression, Claudin-1 upregulation, and the subsequent EMT phenotype (i.e., restoration of E-cadherin and reduction of N-cadherin and Vimentin). Representative blots from two independent biological replicates (n=2) are shown.

**Fig. S4.** The protein expression levels of HIF-1α, Claudin-1, and EMT-related markers in HN6 cells transfected with a HIF-1α overexpression vector and treated with CAP were analyzed by western blot. Experiments were performed with two independent biological replicates (n=2).

**Fig.S5.** Claudin-1 knockdown reverses CAP-mediated EMT suppression. The HN6 cell were transfected with shClaudin-1(5'-GTCTTTGACTCCTTGCTGA-3') or shCON and treated with CAP. Protein expression was analyzed by western blot. β-Actin was used as loading control. Claudin-1 deficiency abolished CAP-induced upregulation of E-cadherin and downregulation of N-cadherin and Vimentin. Shown are representative blots from two independent biological replicates.
