# Supplementary material for: Capsaicin suppresses metastasis in tongue squamous cell carcinoma by reversing epithelial-mesenchymal transition via the AMPK/HIF-1α/Claudin-1 axis
Source: Biol Direct. 2026 Aug 11;21:232. doi: 10.1186/s13062-026-00938-6 (PMC13746642; doi:10.1186/s13062-026-00938-6)
Supplement: Supplementary file 2 [file 13062_2026_938_MOESM13_ESM.docx]

**Table S1. Differences in protein expression between adjacent normal tissues and TSCC of different differentiation grades**

|  | **Tissue type** | **Mean difference (tumor vs. normal)** | **95% CI** | ***P* value (Bonferroni)** |
| --- | --- | --- | --- | --- |
| p-AMPK | Adjacent normal | Reference | — | — |
|  | Well differentiated | –0.28 | (–0.64, 0.07) | 0.203 |
|  | Moderately differentiated | –0.27 | (–0.51, –0.03) | **0.022** |
|  | Poorly differentiated | –0.43 | (–0.79, –0.08) | **0.009** |
| p-ACC | Adjacent normal | Reference | — | — |
|  | Well differentiated | –0.13 | (–0.39, 0.13) | 1.000 |
|  | Moderately differentiated | –0.30 | (–0.48, –0.12) | **< 0.001** |
|  | Poorly differentiated | –0.33 | (–0.60, –0.07) | **0.006** |
| HIF-1α | Adjacent normal | Reference | — | — |
|  | Well differentiated | 0.68 | (–0.92, 2.27) | 1.000 |
|  | Moderately differentiated | 1.16 | (0.18, 2.15) | **0.012** |
|  | Poorly differentiated | 2.23 | (0.64, 3.82) | **0.002** |

**Table footnote:** Values represent mean differences in protein expression levels (tumor − adjacent normal). CI, confidence interval. Bonferroni correction was applied for multiple comparisons. Statistically significant differences (*P* < 0.05) are indicated in bold. Negative mean differences indicate lower expression in tumor tissues compared to adjacent normal tissues; positive mean differences indicate higher expression in tumor tissues.

Due to the exploratory nature of this tissue validation study and the limited sample size (n=37), no a priori power calculation was performed. All subgroup analyses by differentiation grade and pathological stage were considered hypothesis-generating. Multiple comparisons were corrected using the Bonferroni method, but given the small subgroup sizes, both type I and type II errors remain possible. Accordingly, effect sizes with 95% confidence intervals are reported alongside p-values, and results should be interpreted with caution.
