# Supplementary material for: Capsaicin suppresses metastasis in tongue squamous cell carcinoma by reversing epithelial-mesenchymal transition via the AMPK/HIF-1α/Claudin-1 axis
Source: Biol Direct. 2026 Aug 11;21:232. doi: 10.1186/s13062-026-00938-6 (PMC13746642; doi:10.1186/s13062-026-00938-6)
Supplement: Supplementary file 3 [file 13062_2026_938_MOESM14_ESM.docx]

**Table S2. Differences in protein expression between adjacent normal tissues and TSCC of different pathological stages**

|  | **Tissue type** | **Mean difference (tumor vs. normal)** | **95% CI** | ***P* value (Bonferroni)** |
| --- | --- | --- | --- | --- |
| p-AMPK | Adjacent normal | Reference | — | — |
|  | Stage I | –0.20 | (–0.58, 0.19) | 1.000 |
|  | Stage II | –0.35 | (–0.64, –0.06) | **0.010** |
|  | Stage III | –0.29 | (–0.64, 0.05) | 0.153 |
|  | Stage IV | –0.37 | (–0.89, 0.15) | 0.425 |
| p-ACC | Adjacent normal | Reference | — | — |
|  | Stage I | –0.16 | (–0.44, 0.13) | 1.000 |
|  | Stage II | –0.26 | (–0.48, –0.05) | **0.008** |
|  | Stage III | –0.37 | (–0.63, –0.12) | **0.001** |
|  | Stage IV | –0.23 | (–0.70, 0.23) | 1.000 |
| HIF-1α | Adjacent normal | Reference | — | — |
|  | Stage I | 1.34 | (–0.20, 2.89) | 0.134 |
|  | Stage II | 1.31 | (–0.01, 2.64) | 0.054 |
|  | Stage III | 1.07 | (–0.48, 2.61) | 0.465 |
|  | Stage IV | 1.43 | (–1.05, 3.90) | 0.946 |

**Table footnote:** Values represent mean differences in protein expression levels (tumor − adjacent normal). CI, confidence interval. Bonferroni correction was applied for multiple comparisons. Statistically significant differences (*P* < 0.05) are indicated in bold. Negative mean differences indicate lower expression in tumor tissues compared to adjacent normal tissues; positive mean differences indicate higher expression in tumor tissues.
